# Anopheline bionomics, insecticide resistance and transnational dispersion in the context of controlling a possible recurrence of malaria transmission in Jaffna city in northern Sri Lanka

**DOI:** 10.1186/s13071-020-04037-x

**Published:** 2020-03-30

**Authors:** Sinnathamby N. Surendran, Tibutius T. P. Jayadas, Annathurai Tharsan, Vaikunthavasan Thiruchenthooran, Sharanga Santhirasegaram, Kokila Sivabalakrishnan, Selvarajah Raveendran, Ranjan Ramasamy

**Affiliations:** 1 Department of Zoology, Faculty of Science, University of Jaffna, Jaffna, Sri Lanka; 2 Department of Geography, Faculty of Arts, University of Jaffna, Jaffna, Sri Lanka; 3 ID-FISH Technology, Milpitas, CA USA

**Keywords:** *Anopheles* malaria vectors, Insecticide resistance, *kdr* mutation, Jaffna, Larval habitats, Malaria control, Mosquito range expansion, Sri Lanka, Tamil Nadu, Transnational mosquito migration

## Abstract

**Background:**

Malaria was eliminated from Sri Lanka in 2013. However, the influx of infected travelers and the presence of potent anopheline vectors can re-initiate transmission in Jaffna city, which is separated by a narrow strait from the malaria-endemic Indian state of Tamil Nadu.

**Methods:**

Anopheline larvae were collected from different habitats in Jaffna city and the susceptibility of emergent adults to DDT, malathion and deltamethrin investigated.

**Results:**

Anopheline larvae were found in wells, surface-exposed drains, ponds, water puddles and water storage tanks, with many containing polluted, alkaline and brackish water. *Anopheles culicifacies*, *An. subpictus*, *An. stephensi* and *An. varuna* were identified in the collections. Adults of the four anopheline species were resistant to DDT. *Anopheles subpictus* and *An. stephensi* were resistant while *An. culicifacies* and *An. varuna* were possibly resistant to deltamethrin. *Anopheles stephensi* was resistant, *An. subpictus* possibly resistant while *An. varuna* and *An. culicifacies* were susceptible to malathion. DNA sequencing showed a L1014F (TTA to TTC) mutation in the IIS6 transmembrane segment of the voltage-gated sodium channel protein in deltamethrin-resistant *An. subpictus—*a mutation previously observed in India but not Sri Lanka.

**Conclusion:**

*Anopheles subpictus* in Jaffna, like *An. stephensi*, may have recently originated in coastal Tamil Nadu. Besides infected overseas travelers, wind- and boat-borne carriage of *Plasmodium*-infected anophelines across the Palk Strait can potentially reintroduce malaria transmission to Jaffna city. Adaptation to diverse larval habitats and resistance to common insecticides in anophelines are identified as potential problems for vector control should this happen. 
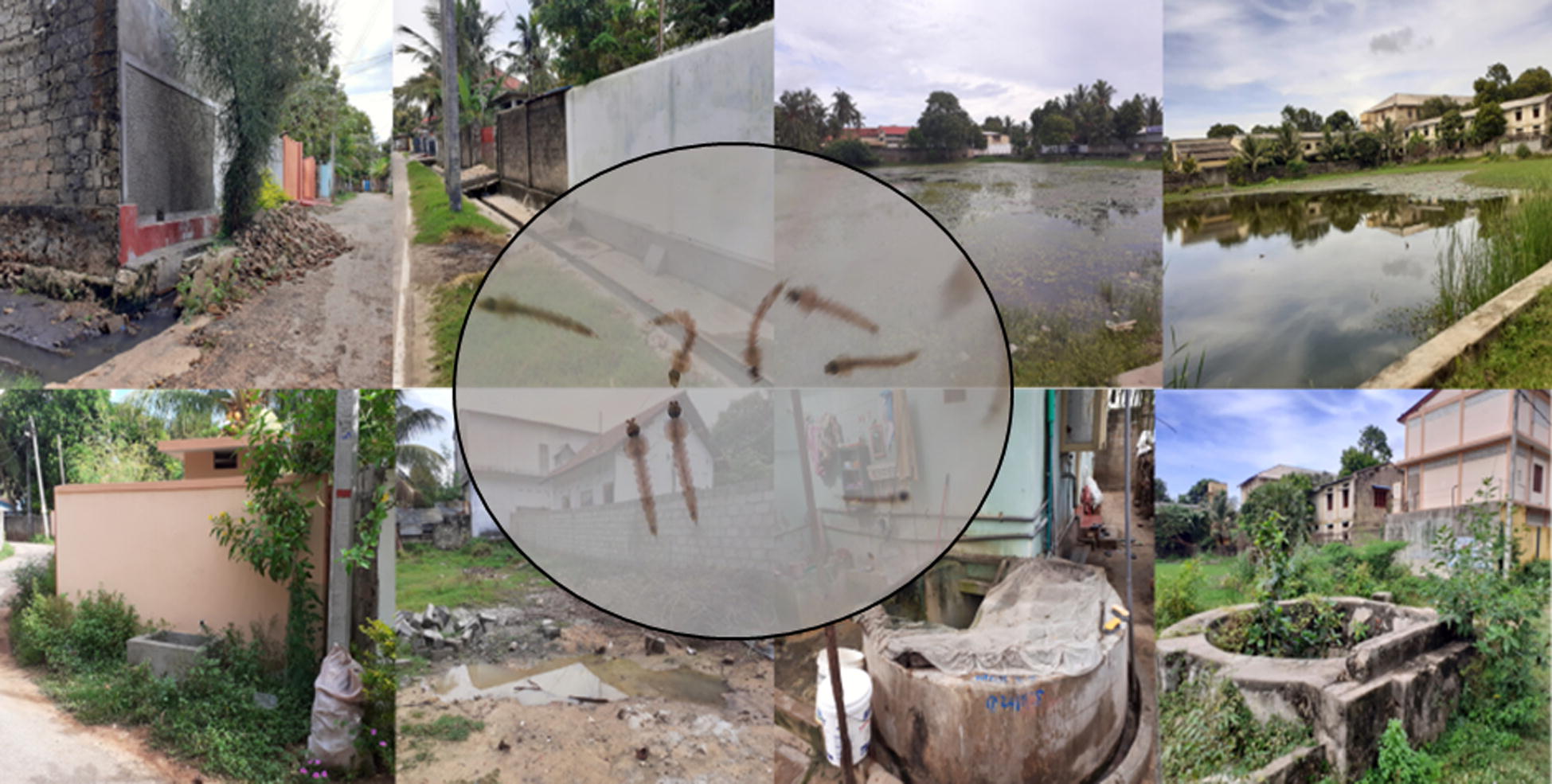

## Background

Malaria remains a global health problem with an estimated 228 million cases and 405,000 deaths worldwide in 2018 [1]. Although the global malaria burden has shown a decreasing trend, and a number of countries have eliminated malaria or are approaching elimination, increasing resistance of vector populations to insecticides in many countries is a cause for concern [1]. Malaria, which had been endemic in Sri Lanka for centuries, was eliminated from the island in 2013. However, the approximately 100 travelers with malaria who arrive every year from endemic countries, and the prevalence of potent anopheline vectors throughout the island, pose the risk of reinitiating local malaria transmission [2]. Indeed, an instance of local malaria transmission from a malaria-infected Indian national to a Sri Lankan resident was documented in late 2018 [3].

The principal malaria vector *Anopheles culicifacies* exists as a species complex in Sri Lanka [4]. Among the two sibling species B and E of the Culicifacies complex, species E was previously considered to be the major vector of malaria in the island [5]. Molecular characterization of *An. subpictus* complex revealed the presence of species A and the morphologically similar *An. sundaicus* (previously classified as *An. subpictus* species B) in Sri Lanka [6]. *Anopheles subpictus* species A and *An. sundaicus* along with *An. annularis* functioned as secondary vectors in Sri Lanka together with several minor vectors including *An. varuna* [7–12]. *Anopheles stephensi* (*sensu stricto*) (type-form), the potent urban vector of malaria in India, was first detected in Sri Lanka in 2017 [13, 14]. The expansion of its range to Sri Lanka occurred after its spread to South India as well as Djibouti, Ethiopia and the Sudan in Northeast Africa [15, 16]. This range expansion was attributed to human migration [15] as well as the adaptation of *An. stephensi* to develop in cement wells and water storage tanks associated with human dwellings, a process exemplifying what was previously termed as the anthropogenically-induced adaptation to invade in mosquito vectors [17].

**Fig. 1:**
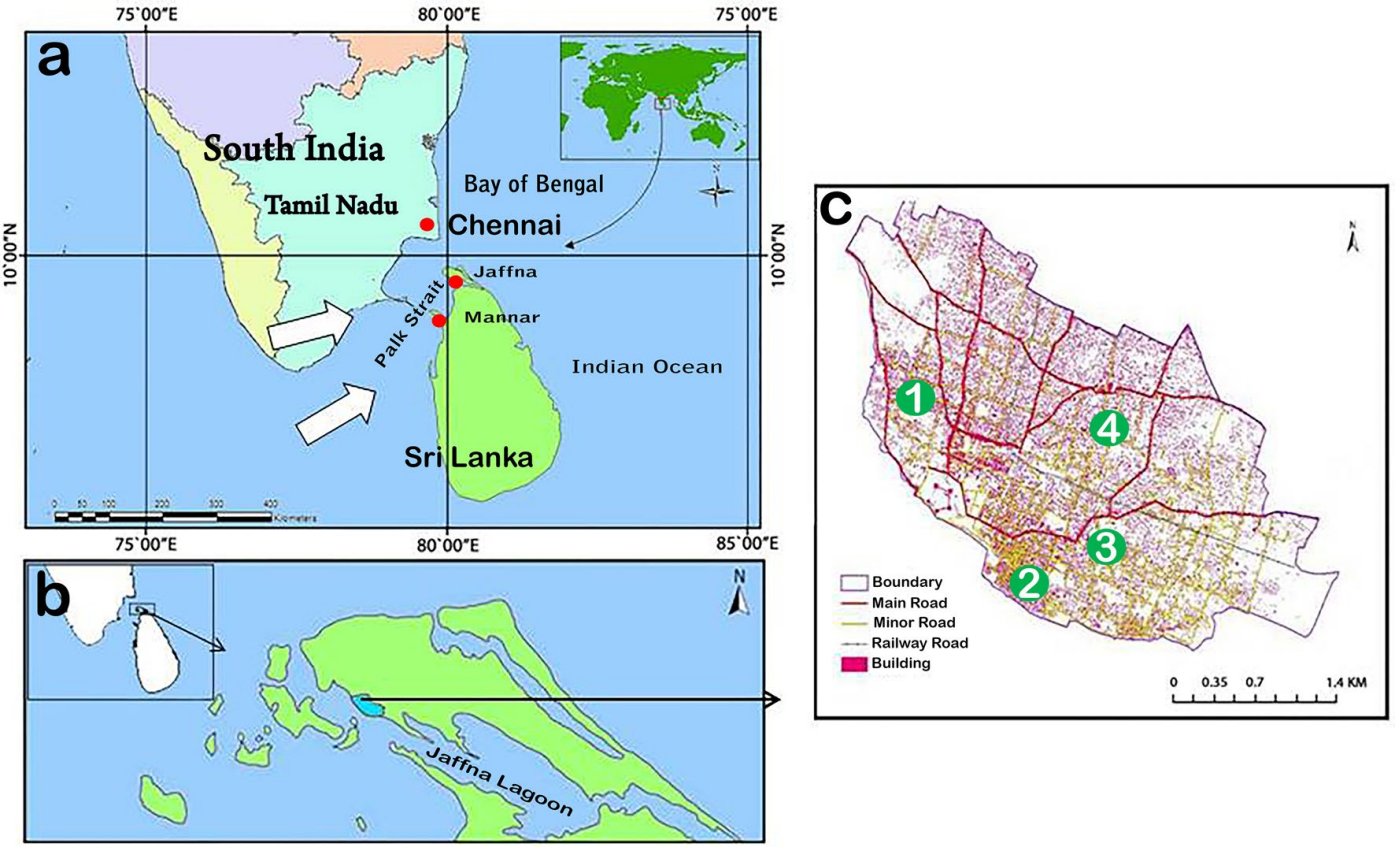
**a** Location of Sri Lanka in the Indian Ocean. Northern Sri Lanka is separated from South India by narrow Palk Strait. Solid white arrows show wind direction during the Southwest monsoon in the Palk Strait. **b** Jaffna city is located in the Jaffna Peninsula of northern Sri Lanka in close proximity to South India. The Jaffna mainland and associated islands are separate by a narrow strip of Jaffna lagoon. **c** Sample collection locations in Jaffna urban area. Green solid circles indicate the four sampled locations *viz*: 1, Kurunagar (9°39′17.5″N, 80°127.05″E); 2, Navanthurai (9°40′16.3″N, 80°029.6″E); 3, Aryalai (9°39′39.9″N, 80°3′1.5″E); and 4, Nallur (9°39′59.99″N, 80°01′60.00″E)

The coastal city of Jaffna (population ~ 100,000, area 10.5 km^2^) is located in the Jaffna Peninsula (population ~ 600,000, area 1130 km^2^) and is the largest city in the Northern Province of Sri Lanka. The Jaffna Peninsula is separated by the 64–137 km wide Palk Strait from Tamil Nadu state in India (Fig. 1a, b). Numerous small inhabited and uninhabited islands lie between the peninsula and Tamil Nadu. Malaria remains endemic in proximal coastal areas of Tamil Nadu [18, 19]. The Jaffna Peninsula is located within the dry zone of Sri Lanka, has a limestone geology and is undergoing rapid salinization of its ground water resources [20, 21]. The salinization is caused by unsustainable rates of water extraction from freshwater aquifers and is exacerbated by rising sea levels. Adaptation of freshwater disease vectors to salinity in coastal areas heightens the potential for transmitting vector-borne diseases as previously described [22]. Malaria control efforts were severely hampered in the Jaffna Peninsula during the civil war of 1983–2009 and malaria vector surveys have not been performed lately in Jaffna city. *Anopheles* species identified recently in the Jaffna peninsula and associated islands include *An. varuna* [23, 24], *An. culicifacies* [23, 24], *An. barbirostris* [23, 25], *An. subpictus* [6, 23, 26], *An. sundaicus* [6, 12, 15, 26, 27] as well as *An. stephensi* [14, 15]. *Anopheles stephensi*, the dominant urban malaria vector in India, was first detected in Mannar island in the Northern Province of Sri Lanka in 2017 (Fig. 1a) [13–15] and subsequently in domestic wells and water storage tanks in Jaffna city in 2018, with a genotype consistent with an origin in Tamil Nadu [15]. Indigenous malaria transmission re-emerging in Jaffna city and the Jaffna Peninsula remains possible because of the proximity to malaria-endemic areas of Tamil Nadu. There is presently no form of authorized sea travel between Jaffna and India but the movement of combatants, refugees and fishermen between Jaffna and Tamil Nadu was common during the 1983–2009 civil war. Characterization of anopheline larvae and their habitats in the Jaffna city and the susceptibility of adults emerging from the collected larvae to common insecticides was undertaken to assess the potential for malaria transmission re-establishing itself in the city.

## Methods

### Larval collection and rearing

**Fig. 2:**
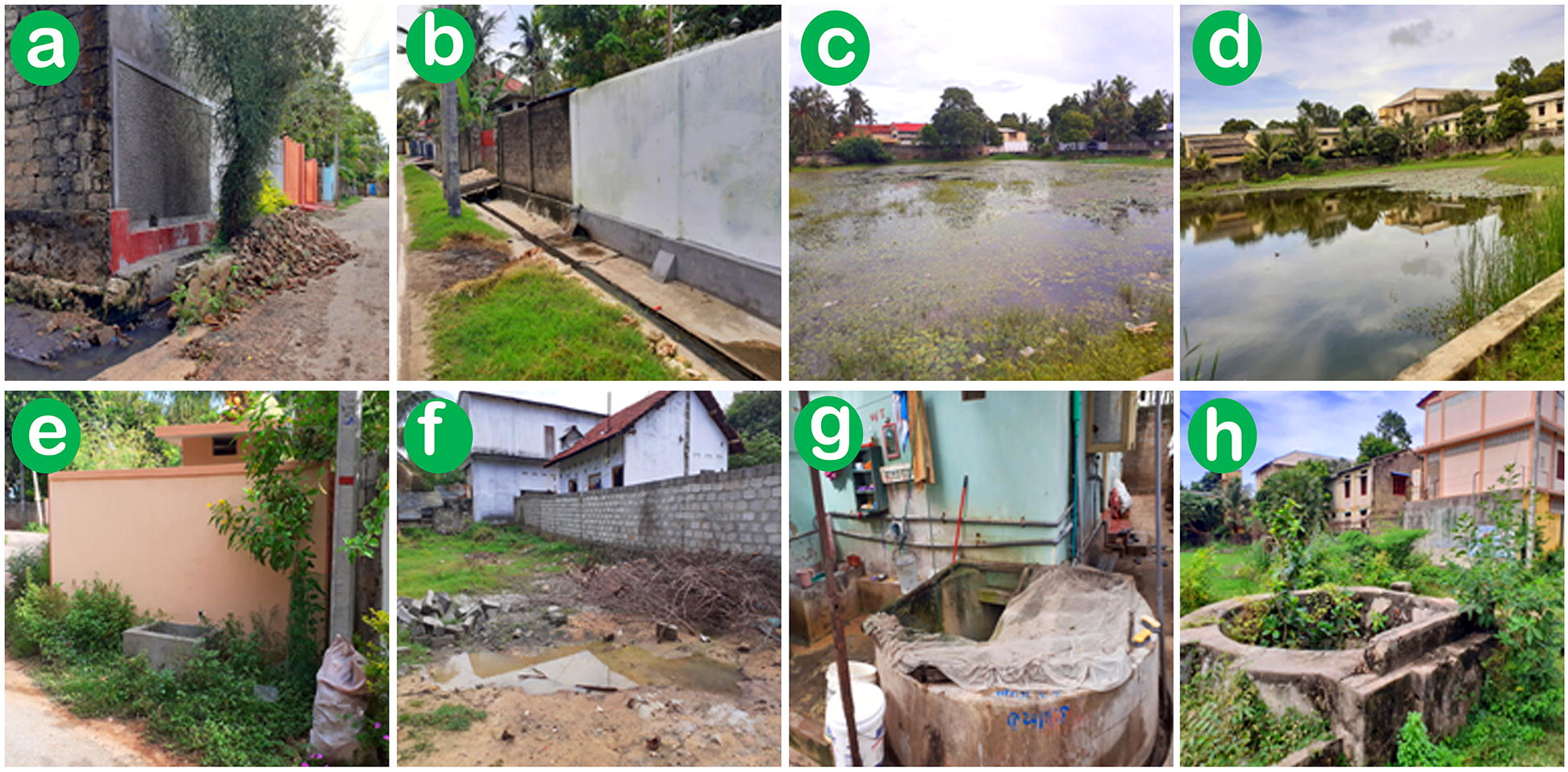
Anopheline larval preimaginal development habitats in Jaffna city. **a**, **b** Open surface drain. **c**, **d** Pond. **e** Water storage tank. **f** Puddle. **g** Domestic well in use. **h** Unused well

Larval sampling was done and water quality in larval habitats determined fortnightly, at four locations in Jaffna city (Fig. 1c) during the period April 2018 to August 2019. Samples were collected from Kurunagar (9°39′17.5″N, 80°127.05″E), Navanthurai (9°40′16.3″N, 80°0′29.6″E), Aryalai (9°39′39.9″N, 80°3’1.5″E) and Nallur (9°39′59.99″N, 80°01′60.00″E). Anopheline larvae were collected from deep wells using string-connected conical drop nets with 15 cm diameter and 10 cm depth. Standard 350 ml dippers were used to collect larvae from puddles, ponds, open surface drains, water storage tanks and shallow wells (Fig. 2) with an average of 10 dips per habitat. The pH, salinity, dissolved oxygen (DO), total dissolved solids (TDS), and electrical conductivity in water collections with anopheline larvae were measured with a Multi Parameter Probe (Hanna Instruments, Leighton Buzzard, UK). Larval density for each species was calculated as the number of larvae per liter for every collection site. Habitats where anopheline larvae were not found were not analyzed for water characteristics or statistical studies.

Collected larvae were reared as described previously in the insectary of the Department of Zoology, University of Jaffna, Jaffna, Sri Lanka, to reach adulthood [26] in water from their respective habitats. Emergent adults were identified morphologically using published keys [28, 29] and used for insecticide bioassays when 3–5 days-old.

### Insecticide susceptibility tests

Insecticide susceptibility tests were performed according to the standard WHO protocol for adult mosquitoes [30] as previously described [31]. Adults of each species from all collection sites were pooled and exposed to insecticide impregnated papers of 0.05% (w/v) deltamethrin, 5% (w/v) malathion and 4% (w/v) DDT using WHO bioassay kits and procedure [30]. Mosquitoes were exposed for 1 h and the mortalities determined after a 24 h recovery period. Mosquitoes were exposed to each insecticide in two or three replicates in two separate experiments. Control mosquitoes were exposed to papers impregnated with carrier oil alone in each experiment. Mortality was determined and adjusted using Abbot’s formula if the control mortalities were between 5–20% [30, 32]. The WHO criteria were used to define a population as susceptible (mortality of > 98%), possibly resistant (mortality of 90–97%) and resistant (mortality of < 90%) [30]. Mosquitoes that survived exposure to deltamethrin in the bioassay were frozen individually at − 20 °C to screen for knock-down resistance (*kdr*) mutations in the voltage-gated sodium channel protein (VGSC).

### DNA sequencing to differentiate *An. subpictus* and *An. sundaicus*

One hundred and fifty adult *An. subpictus* comprising approximately 50 mosquitoes that were tested for each insecticide were randomly selected for differentiation from the morphologically similar *An. sundaicus* by the previously described diagnostic allele-specific PCR [6].

### DNA sequencing of the voltage-gated sodium channel protein gene

Twenty each of deltamethrin-resistant *An. subpictus* and *An. stephensi* were used to PCR amplify the IIS6 transmembrane coding segment of the *VGSC* gene using the primers KdrF (5′-GGA CCA YGA TTT GCC AAG ATG-3′) and KdrR (5′-CGA AAT TGG ACA AAA GCA AAG-3′ [33]. The PCR products were purified using QIAquick® PCR Purification Kit (Qiagen, California, USA) and sequenced in both directions at Macrogen, Seoul, South Korea. The sequences were edited in Finch TV (Geospiza, Seattle, USA) and aligned with Clustal W in MEGA 5.0 software [34].

### Statistical analysis

A one-way ANOVA was performed to determine associations between the different types of habitats where anopheline larvae were found and each of the measured water parameters separately. Since the sample size was small for each species type of habitat data, and similar habitats where larvae were absent were excluded from the study, the species-type of habitat interaction was not statistically analysed. The influence of water characteristics on the density of each species of anopheline larvae in different habitats where the larvae were present was analysed using generalised linear mixed model (GLMM) with habitats as a unique identifier. The larval densities were fitted to a negative binomial distribution. The total model was represented as, physical parameters / larvae density = species/solution (effect of water parameters). The logistic regression model was used in the analysis. From the effect of water parameters on overall species obtained from the total model, least square means were compared to understand the effect on each species. The value of *P* < 0.05 was considered statistically significant. Statistical analyses were performed using the SAS University Edition (SAS Institute Inc., NC, USA).

## Results

### Anopheline species and water habitat characteristics in preimaginal habitats

A total of 2369 larvae of four *Anopheles* species were collected from five different types of habitats (Table 1). A total of 2279 adults emerged from the larval collections and were identified morphologically as *Anopheles subpictus*, *An. stephensi*, *An. culicifacies* and *An. varuna*.

**Table 1:** Anopheline larvae collected from different habitats

Species	Number of larvae collected in different habitats					Total (%)
	Open surface drain^a,^^b^	Pond^c, d^	Water storage tank^e^	Puddle^f^	Well^g, h^	
*An. stephensi*	–	–	120	–	642	762 (33.5)
*An. subpictus*	143	141	–	43	296	623 (27.3)
*An. culicifacies*	98	83	–	28	167	376 (16.5)
*An. varuna*	190	12	35	–	281	518 (22.7)
Total	431	236	155	71	1386	2279

*Note*: Superscript letters ^a–h^ refer to the images of the corresponding habitats in Fig. 2

*Anopheles subpictus*, *An. culicifacies* and *An. varuna* were present in wells (number of wells, *n* = 35), open water storage tanks (*n* = 10), open surface drains (*n* = 9), puddles (*n* = 3) and ponds (*n* = 9), in all four locations but *An. stephensi* was only collected in wells (*n* = 14) and water storage tanks (*n* = 2), as shown in Table 1 in three out of four locations: Gurunagar (wells and water storage tank); Navanthurai (wells); and Nallur (wells and water storage tank). Larvae of *An. culicifacies*, *An. subpictus* and *An. varuna* were often found to occur together in the same habitat. However, other anopheline species were not found in the limited number of water tanks and wells where *An. stephensi* larvae were present. Water parameters in the different habitats where larvae were collected were quite variable (Table 2, Fig. 2). ANOVA revealed no significant differences in the mean pH of the habitats where anopheline larvae were collected (*F*_(4, 61)_ = 0.743, *P* = 0.55). However, the other water parameters varied significantly between the different habitats where anopheline larvae were present: DO (*F*_(4, 61)_ = 7.761, *P* < 0.001), conductivity (*F*_(4, 61)_ = 7.666, *P* < 0.001), TDS (*F*_(4, 61)_ = 2.551, *P* = 0.048) and salinity (*F*_(4, 61)_ = 2.701, *P* = 0.038). It was apparent that all four anopheline species could oviposit and undergo preimaginal development to adults in relatively alkaline, brackish and polluted water in Jaffna city. The GLMM analysis showed that larval densities of each of the four anopheline species were significantly (*t* ranging from -4.38 to -7.67; *P* = 0.001 to 0.008) and negatively associated with salinity but not significantly (*P* > 0.05) associated with pH, TDS, DO or conductivity of the habitats where the respective species of anopheline larvae were present (Additional file 1: Table S1)

**Table 2:** Characteristics of water habitats associated with the different species of anopheline larvae

Parameter	Parameter range in the habitats where larvae were found (mean ± SD)			
	*An. stephensi*	*An. subpictus*	*An. culicifacies*	*An. varuna*
pH	6.5–8.9 (7.3 ± 0.7)	6.6–8.9 (7.6 ± 0.6)	6.8–8.9 (7.4 ± 0.6)	6.6–8.9 (7.2 ± 0.5)
Salinity (ppt)	1.9–3.4 (2.2 ± 0.4)	0.9–3.4 (1.6 ± 0.7)	0.9–3.4 (1.5 ± 0.7)	0.5–1.4 (1.2 ± 0.5)
TDS (ppm)	1730–3212 (1948 ± 418)	951–3112 (1791 ± 646)	951–3112 (1691 ± 586)	482–1982 (1324 ± 583)
DO (ppm)	4.3–5.5 (4.8 ± 0.4)	3.9–5.5 (5.1 ± 0.6)	4.3–6.0 (5.0 ± 0.7)	3.9–6.0 (4.9 ± 0.6)
Conductivity (µS/cm)	3449–6202 (3678 ± 973)	1899–4743 (3339 ± 1061)	1899–4122 (2915 ± 891)	964–4743 (2534 ± 1041)

*Notes*: The results show the range, mean and standard deviation of the mean (sd) of measured parameters. Freshwater and brackish water are defined as containing < 0.5 and 0.5–30 ppt salinity respectively [38, 39]. Water with ≥ 400 ppm TDS, ≤ 4.5 ppm DO and ≥ 800 μS/cm conductivity are indicative of pollution [58]

*Abbreviations*: TDS, total dissolved solids; DO, dissolved oxygen; ppt, parts per thousand; ppm, parts per million; µS/cm, micro Siemens per centimetre; SD, standard deviation

### Adult insecticide susceptibility bioassays

The results of the insecticide susceptibility bioassays according to the WHO criteria are presented in Table 3 (see also Additional file 2: Table S2). All four anopheline species collected in Jaffna city were resistant to DDT. *Anopheles subpictus* and *An. stephensi* were resistant, while *An. culicifacies* and *An. varuna* were possibly resistant to the widely used pyrethroid deltamethrin. The malathion bioassay showed that *An. stephensi* was resistant; *An. subpictus* can be considered resistant as its 95% confidence limits for resistance were 89–91%; and both *An. culicifacies* and *An. varuna* were susceptible.

**Table 3:** Insecticide susceptibility of the different anopheline species

Insecticide	*An. stephensi*		*An. subpictus*		*An. varuna*		*An. culicifacies*	
	% mortality	95% CI	% mortality	95% CI	% mortality	95% CI	% mortality	95% CI
DDT 4%	^R^14 ± 5 (100)	12–15	^R^2 ± 3 (100)	1–3	^R^39 ± 1 (100)	38–39	^R^22 ± 5 (100)	21–23
Malathion 5%	^R^34 ± 9 (110)	3–36	^V^90 ± 1 (100)	89–91	^S^100 (100)	100	^S^98 ± 2 (100)	97–99
Deltamethrin 0.05%	^R^56 ± 1 (100)	55–57	^R^10 ± 5 (110)	9–11	^V^97 ± 3 (102)	96–98	^V^96 ± 5 (106)	95–97

*Notes*: Data represent mean percent mortality ± SD (*n*). Detailed data are shown in Additional file 2: Table S2

*Abbreviations*: *n*, total number of mosquitoes tested; S, susceptible (≥ 98% mortality); R, confirmed resistance (< 90% mortality); V, possible resistance and verification needed (90–97% mortality); SD, standard deviation; CI, confidence interval

Of the *An. subpictus* mosquitoes that were resistant to the three insecticides, 150 were screened by PCR and all were confirmed to be *An. subpictus* species A and not *An. sundaicus*.

### Sequence analysis of the *VGSC* gene

We sequenced the IIS6 coding domain of the *VGSC* gene in *An. subpictus* and *An. stephensi* that were resistant to deltamethrin. Fifteen sequences, of approximately 250 bp in size, of each species were analyzed. This revealed the presence of the same A to C transversion leading to a L1014F (TTA to TTC) amino acid substitution in all 15 samples of *An. subpictus* (Additional file 3: Alignment S1). Fourteen of the 15 mosquitoes were homozygous for this L1014F mutation. No mutations were observed in the sequenced region of the *VGSC* gene in *An. stephensi*. The mutated sequence found in the 15 *An. subpictus* (accession number MK249732) and wild type sequences found in the 15 *An. stephensi* (accession number MK248685) have been deposited with GenBank.

## Discussion

*Anopheles culicifacies* had been widely held to only undergo preimaginal development in unpolluted freshwater habitats in rural Sri Lanka [9, 35]. However, recent studies have shown that it is able to develop in brackish water of 2–4 ppt salinity in the Jaffna Peninsula and its offshore Island Delft [23] and in the Eastern Province [36]. *Anopheles culicifacies* larvae have also been found in polluted drains with low DO content in the Eastern Province [37]. Our study suggests that it can undergo preimaginal development to adulthood in polluted water with high TDS (> 400 ppm) and low DO (≤ 4.5 ppm) and relatively alkaline water in Jaffna city. The ability to tolerate brackish, polluted and alkaline water in Jaffna city was also observed with *An. subpictus* (identified here as being mostly if not exclusively as *An. subpictus* species A and distinct from the closely-related, salinity-tolerant *An. sundaicus*) which had also been previously regarded as developing only in fresh and unpolluted water [38]. Although we had previously observed *An. stephensi* to tolerate brackish water of up to 3.5 ppt salinity in Jaffna city [15] which is consistent with the present findings, we now show that it can also develop in polluted and alkaline water. Because of its likely recent arrival from coastal Tamil Nadu, it is possible that tolerance to brackish and polluted water in *An. stephensi* are characteristics developed in Tamil Nadu or elsewhere in India that have helped it expand its range to Jaffna in an example of the anthropogenically-induced adaptation to invade in mosquito vectors [15, 17].

We also found that *An. varuna*, previously regarded as another freshwater mosquito [35], is capable of developing in brackish water of up to 1.4 ppt salinity as well as polluted and alkaline water in Jaffna city. Ground water is relatively alkaline throughout the Jaffna Peninsula because of its limestone geology [21]. Ground water is becoming increasingly brackish in the Jaffna Peninsula, due to an unsustainable depletion of its freshwater limestone aquifers and influx of sea water [21, 39]. These factors may have promoted adaptation to alkalinity and salinity in the four anophelines in Jaffna city. Rising sea levels exacerbating salinization of ground water in the peninsula [21, 22] can further increase salinity tolerance in mosquito vectors in the future.

However, the statistical analysis of the relationship between larval density and habitat salinity also suggest that the four anopheline species in Jaffna city have still retained a preference for habitats of lower salinity for laying eggs and undergoing preimaginal development. Preimaginal development in brackish water habitats in Jaffna city and elsewhere in the Jaffna Peninsula was also recently documented in the typical freshwater arboviral vectors *Aedes aegypti* and *Ae. albopictus* [23, 40]. Salinity adaptation in *Ae. aegypti* has been shown to involve heritable and non-heritable biological changes [41, 42]. The ability of *Ae. aegypti* to develop in polluted water in surface drains in Jaffna city is associated with greater resistance to pyrethroids [43]. Elucidating biological changes in anophelines associated with similar adaptation to salinity and pollution in Jaffna city can further help understand the adaptability of different mosquitoes to anthropogenic environmental changes. Furthermore, our results suggest that mosquito larval control measures in the city that are presently directed almost exclusively towards fresh water habitats, need to be extended to brackish and polluted water habitats. This is presently important for *Ae. aegypti* and *Ae. albopictus* because dengue and chikungunya are endemic in Jaffna [44] but can become essential also for anopheline vector control should malaria transmission recur in Jaffna.

The four anopheline species collected in Jaffna city were resistant to DDT despite the cessation of DDT use for indoor residual spraying (IRS) in Jaffna in early 1970s. Resistance to DDT declined slowly after cessation of use, but increased again after 1983 due to a glutathione-S transferase-based mechanism, postulated to be initially caused by DDT and subsequently by exposure to the malathion used for IRS and other organophosphates used for controlling agricultural pests [2, 27, 45, 46]. The results confirm that *An. stephensi* in Jaffna is resistant to all three insecticides tested consistent with our recent previous report [15]. *Anopheles culicifacies* and *An. varuna* were susceptible to malathion but both, together with *An. subpictus*, showed varying levels of resistance to deltamethrin. Resistance to deltamethrin in *Ae. aegypti* developing in polluted drain water in Jaffna city was suggested to be caused by adaptation to cope with organic pollutants in drain water and possibly the common use of pyrethroids to control adult mosquitoes and other insect pests in Jaffna [43]. The same factors may also be responsible for deltamethrin resistance in Jaffna city anophelines.

Pyrethroids and DDT target the *VGSC* to alter ion transport in arthropod nerve cell membranes [47, 48]. This leads to paralysis and eventual death of the insect. Mutations in *VGSC*, prominently L1014F, confer knock-down resistance (*kdr*) against pyrethroids and DDT in many anopheline species worldwide [47]. In *An. subpictus*, an L1014F mutation is reported to arise from an A → T transversion in the North Central Province of Sri Lanka [49] and India [50] and an A → C transversion only in India [50]. Only one of 25 etofenprox resistant *An. subpictus* carried the A → T transversion and none carried the A → C transversion in Sri Lanka [49]. In contrast the frequencies of A → T and A → C transversions were 0.19 and 0.67 respectively in deltamethrin-resistant Indian *An. subpictus* [50]. The A→C transversion that leads to the L1014F was found in Indian *An. subpictus* that had 100% genetic similarity with Sri Lankan *An. subpictus* species A in the mitochondrial cytochrome oxidase I gene [50]. Our results show that an A → C transversion that leads to the L1014F amino acid substitution is found in all 15 *An. subpictus* sequenced in Jaffna city. To our knowledge, this is the first report of an A → C transversion leading to an L1014F mutation in *An. subpictus* sibling species A in Sri Lanka, and it raises the possibility that deltamethrin-resistant *An. subpictus* in Jaffna city, like *An. stephensi* [15], could have originated in India rather than Sri Lanka.

Although the recently arrived *An. stephensi* in Jaffna was resistant to DDT and deltamethrin [15], we now show that there are no mutations in the sequenced IIS6 segment of the VGSC protein in this mosquito. Resistance may, therefore, be caused by other mechanisms such as increased activities of detoxifying enzymes [27, 45, 46] and this merits further investigation.

Mosquito vectors have been regarded as migrating at most a few kilometers after taking a blood meal [51, 52] and therefore *Plasmodium*-infected mosquitoes were thought incapable of traversing the Palk Strait separating the Jaffna Peninsula from coastal Tamil Nadu (Fig. 1). However, a recent study in the Sahel region of Africa provided evidence that blood-fed anopheline vectors can disperse > 100 km aided by winds of < 10 m/s [53]. The wind action over the Palk Bay, Bay of Bengal, India and Sri Lanka is mainly influenced by two monsoons, the Southwest (May to July) and Northeast (October to January). The average wind speed during the Southwest monsoon is ~5–7 m/s and during this monsoon the wind has a strong component blowing from coastal Tamil Nadu towards the Jaffna Peninsula (arrowed in Fig. 1a) [54]. Data from the Conformal Cubic Atmospheric Model (CCAM) simulation over Sri Lanka [55] predicts that some wind during the Southwest monsoon period may be deflected in a more easterly direction towards Jaffna because of the Western Ghats mountain range (Fig 1a). We therefore propose that wind-borne migration of anophelines is possible from coastal areas of Tamil Nadu to the Jaffna Peninsula and Mannar Island, and this might explain the genetic characteristics of *An. subpictus* and *An. stephensi* in Jaffna city. However, genetic characterization at multiple genetic loci in *An. subpictus* from Jaffna city and many different locations in Tamil Nadu and elsewhere in Sri Lanka are needed to establish this hypothesis for *An. subpictus.* We had previously suggested that *An. stephensi* from India may have arrived as eggs/larvae in water collections in boats used by fishermen and combatants during the 1983–2009 civil war [15]. Arrival as eggs/larvae in boats is a possibility for both *An. subpictus* and *An. stephensi*, but wind-borne and boat-borne migration of adult female anophelines are important new possibilities for consideration. The wind-aided arrival of female anophelines that have taken a *Plasmodium*-infected blood meal in Tamil Nadu could readily re-initiate malaria transmission in locations such as Jaffna city because of the high population density and the presence of potent local vectors. Wind-borne movement of *Plasmodium*-infected anophelines across national borders is therefore an additional factor for consideration in malaria control and elimination efforts in other parts of the world [2, 15] including islands [39].

Currently, the regional Anti-malaria Campaign of the Jaffna District applies temophos to unused wells and introduces larvivorous fish to domestic wells to control preimaginal development of anophelines in Jaffna city. We have previously shown that the larvivorous fish *Oreochromis mossambicus* readily feeds on *An. subpictus* [56], and the introduction of *Poecilia reticulata* to domestic wells and water tanks is effective in controlling *An. stephensi* in Jaffna [15]. In the face of widespread insecticide resistance, the introduction of larvivorous fish into wells and water storage tanks may contribute to controlling anopheline populations in Jaffna city.

A limitation of our study is that the *An. culicifacies* collected in Jaffna city was not identified as being species B or species E due to the difficulty in karyotyping and the lack of differentiating DNA markers [57]. More extensive sampling of habitats where larvae are present or absent in Jaffna city is also needed for firmly associating the types of habitats and water characteristics with species abundance. Both entailed a larger project than was possible in the present study.

## Conclusions

Vector control plays an important role in limiting the spread of malaria. Our findings highlight resistance to common insecticides and the adaptation of anopheline vectors to polluted, brackish and alkaline water habitats in Jaffna city. These factors together with arrival of infected travelers and the possible wind-borne carriage of malaria-infected anophelines from Tamil Nadu are elements to consider for maintaining Jaffna city and mainland Sri Lanka free from malaria.

## Supplementary information

**Additional file 1: Table S1.** Association of water parameters with the density of anopheline larvae in the different habitats where anopheline larvae were present.

**Additional file 2: Table S2.** Details of insecticide susceptibility tests.

**Additional file 3: Alignment S1.** DNA sequence alignments of *NaV* region.

## Data Availability

All data generated during this study are included in this published article and its additional files. Voucher specimens of identified anopheline mosquitoes were deposited in the Zoology Museum of the Department of Zoology, University of Jaffna. The newly generated DNA sequences were deposited in the GenBank database under the accession numbers MK249732, MK249733 and MK248685.

## Abbreviations

AMC: anti-malaria campaign
DDT: dichlorodiphenyltrichloroethane
DO: dissolved oxygen
IRS: indoor residual spraying
VGSC: voltage-gated sodium channel
PCR: polymerase chain reaction
ppt: parts per thousand
ppm: parts per million
TDS: total dissolved solids
WHO: World Health Organization
