# Supplementary material for: Anopheline bionomics, insecticide resistance and transnational dispersion in the context of controlling a possible recurrence of malaria transmission in Jaffna city in northern Sri Lanka
Source: Parasit Vectors. 2020 Mar 30;13:156. doi: 10.1186/s13071-020-04037-x (PMC7106892; doi:10.1186/s13071-020-04037-x)
Supplement: Supplementary file 1 — Additional file 1: Table S1. Association of water parameters with the density of anopheline larvae in the different habitats where anopheline larvae were present. [file 13071_2020_4037_MOESM1_ESM.docx]

**Additional file 1:** **Table S1.** Association of water parameters with the density of anopheline larvae in the different habitats where anopheline larvae were present

| Parameters | Species | GLMM negative binomial model | | | | |
| --- | --- | --- | --- | --- | --- | --- |
|  |  | *β* | SE | DF | *t value* | *P value* |
| pH | *An. stephensi* | 0.2368 | 0.2572 | 3 | 0.92 | 0.455 |
|  | *An. subpictus* | 1.1375 | 0.5591 | 3 | 2.03 | 0.179 |
|  | *An. varuna* | 0.3553 | 0.3320 | 3 | 1.07 | 0.397 |
|  | *An. culicifacies* | 0.2906 | 0.4227 | 3 | 0.69 | 0.563 |
| Salinity | *An. stephensi* | -1.5347 | 0.2 | 3 | -7.67 | 0.001* |
|  | *An. subpictus* | -0.9343 | 0.2133 | 3 | -4.38 | 0.003* |
|  | *An. varuna* | -1.6695 | 0.2345 | 3 | -7.12 | 0.002* |
|  | *An. culicifacies* | -1.6216 | 0.2880 | 3 | -5.63 | 0.008* |
| Dissolved oxygen | *An. stephensi* | -0.2879 | 0.2443 | 3 | -1.18 | 0.324 |
|  | *An. subpictus* | 1.0227 | 0.3120 | 3 | 3.28 | 0.07 |
|  | *An. varuna* | 0.2957 | 0.2634 | 3 | 1.12 | 0.343 |
|  | *An. culicifacies* | 0.0847 | 0.3538 | 3 | 0.24 | 0.826 |
| Total dissolved solids | *An. stephensi* | 0.9784 | 0.4486 | 3 | 2.18 | 0.312 |
|  | *An. subpictus* | 0.0243 | 0.4146 | 3 | 1.06 | 0.180 |
|  | *An. varuna* | 0.6858 | 0.4323 | 3 | 1.59 | 0.115 |
|  | *An. culicifacies* | 1.3422 | 0.8003 | 3 | 1.68 | 0.114 |
| Conductivity | *An. stephensi* | 0.2364 | 0.3453 | 3 | 0.68 | 0.552 |
|  | *An. subpictus* | 0.3345 | 0.4150 | 3 | 1.46 | 0.457 |
|  | *An. varuna* | 2.3514 | 0.7400 | 3 | 2.18 | 0.188 |
|  | *An. culicifacies* | 1.3558 | 0.2546 | 3 | 1.22 | 0.354 |

**Legend to Additional File 1.** *β –* estimation*,* SE – standard error, DF – degree of freedom, * - significant at P<0.05
