# Supplementary material for: Anopheline bionomics, insecticide resistance and transnational dispersion in the context of controlling a possible recurrence of malaria transmission in Jaffna city in northern Sri Lanka
Source: Parasit Vectors. 2020 Mar 30;13:156. doi: 10.1186/s13071-020-04037-x (PMC7106892; doi:10.1186/s13071-020-04037-x)
Supplement: Supplementary file 2 — Additional file 2: Table S2. Details of insecticide susceptibility tests. [file 13071_2020_4037_MOESM2_ESM.docx]

**Additional file 2:** **Table S2**. Details of insecticide susceptibility tests.

| **Species** | **DDT 4%** | | | | **DDT control** | | | | **malathion 5%** | | | | **malathion control** | | | | **deltamethrin 0.05%** | | | | | **deltamethrin control** | | | | |
| --- | --- | --- | --- | --- | --- | --- | --- | --- | --- | --- | --- | --- | --- | --- | --- | --- | --- | --- | --- | --- | --- | --- | --- | --- | --- | --- |
| ***An. stephensi*** | Replicate | T | S | M | Replicate | T | S | M | Replicate | T | S | M | Replicate | T | S | M | Replicate | T | S | M | Replicate | | T | S | M |  |
|  | 1R1 | 17 | 11 | 6 | 1C | 10 | 10 | 0 | 1R1 | 20 | 14 | 6 | 1C | 12 | 12 | 0 | 1R1 | 20 | 11 | 9 | 1C | | 16 | 16 | 0 |  |
|  | 1R2 | 15 | 13 | 2 |  |  |  |  | 1R2 | 16 | 4 | 12 |  |  |  |  | 1R2 | 20 | 6 | 14 |  | |  |  |  |  |
|  | 1R3 | 19 | 18 | 1 |  |  |  |  | 1R3 | 20 | 15 | 5 |  |  |  |  | 1R3 | 20 | 10 | 10 |  | |  |  |  |  |
|  | 2R1 | 19 | 19 | 0 | 2C | 12 | 12 | 0 | 2R1 | 19 | 13 | 6 | 2C | 14 | 14 | 0 | 2R1 | 20 | 7 | 13 | 2C | | 8 | 8 | 0 |  |
|  | 2R2 | 30 | 24 | 6 |  |  |  |  | 2R2 | 15 | 12 | 3 |  |  |  |  | 2R2 | 20 | 10 | 10 |  | |  |  |  |  |
|  |  |  |  |  |  |  |  |  | 2R3 | 20 | 15 | 5 |  |  |  |  |  |  |  |  |  | |  |  |  |  |
| ***An. subpictus*** |  |  |  |  |  |  |  |  |  |  |  |  |  |  |  |  |  |  |  |  |  | |  |  |  |  |
|  | 1R1 | 20 | 20 | 0 | 1C | 14 | 14 | 0 | 1R1 | 20 | 4 | 16 | 1C | 20 | 20 | 0 | 1R1 | 16 | 16 | 0 | 1C | | 10 | 10 | 0 |  |
|  | 1R2 | 17 | 14 | 1 |  |  |  |  | 1R2 | 20 | 2 | 18 |  |  |  |  | 1R2 | 20 | 17 | 3 |  | |  |  |  |  |
|  | 1R3 | 16 | 14 | 1 |  |  |  |  | 1R3 | 17 | 0 | 17 |  |  |  |  | 1R3 | 20 | 19 | 1 |  | |  |  |  |  |
|  | 2R1 | 16 | 16 | 0 | 2C | 16 | 16 | 0 | 2R1 | 17 | 2 | 15 | 2C | 15 | 15 | 0 | 2R1 | 20 | 15 | 5 | 2C | | 10 | 10 | 0 |  |
|  | 2R2 | 18 | 18 | 0 |  |  |  |  | 2R2 | 26 | 3 | 23 |  |  |  |  | 2R2 | 28 | 27 | 1 |  | |  |  |  |  |
|  | 2R3 | 13 | 13 | 0 |  |  |  |  |  |  |  |  |  |  |  |  |  |  |  |  |  | |  |  |  |  |
| ***An. culicifacies*** |  |  |  |  |  |  |  |  |  |  |  |  |  |  |  |  |  |  |  |  |  | |  |  |  |  |
|  | 1R1 | 14 | 13 | 1 | 1C | 12 | 12 | 0 | 1R1 | 20 | 2 | 18 | 1C | 8 | 8 | 0 | 1R1 | 20 | 1 | 19 | 1C | | 8 | 8 | 0 |  |
|  | 1R2 | 18 | 14 | 4 |  |  |  |  | 1R2 | 20 | 0 | 20 |  |  |  |  | 1R2 | 20 | 2 | 18 |  | |  |  |  |  |
|  | 1R3 | 19 | 14 | 5 |  |  |  |  | 1R3 | 20 | 0 | 20 |  |  |  |  | 1R3 | 16 | 1 | 15 |  | |  |  |  |  |
|  | 2R1 | 16 | 13 | 3 | 2C | 8 | 8 | 0 | 2R1 | 28 | 0 | 28 | 2C | 8 | 8 | 0 | 2R1 | 16 | 0 | 16 | 2C | | 8 | 8 | 0 |  |
|  | 2R2 | 20 | 15 | 5 |  |  |  |  | 2R2 | 12 | 0 | 12 |  |  |  |  | 2R2 | 18 | 0 | 18 |  | |  |  |  |  |
|  | 2R3 | 13 | 9 | 4 |  |  |  |  |  |  |  |  |  |  |  |  | 2R3 | 16 | 0 | 16 |  | |  |  |  |  |
| ***An. varuna*** |  |  |  |  |  |  |  |  |  |  |  |  |  |  |  |  |  |  |  |  |  | |  |  |  |  |
|  | 1R1 | 18 | 9 | 9 | 1C | 14 | 14 | 0 | 1R1 | 20 | 0 | 20 | 1C | 16 | 16 | 0 | 1R1 | 30 | 2 | 28 | 1C | | 15 | 15 | 0 |  |
|  | 1R2 | 28 | 17 | 11 |  |  |  |  | 1R2 | 20 | 0 | 20 |  |  |  |  | 1R2 | 15 | 0 | 15 |  | |  |  |  |  |
|  | 1R3 | 20 | 15 | 5 |  |  |  |  | 1R3 | 20 | 0 | 20 |  |  |  |  | 1R3 | 16 | 1 | 15 |  | |  |  |  |  |
|  | 2R1 | 20 | 13 | 7 | 2C | 12 | 12 | 0 | 2R1 | 20 | 0 | 20 | 2C | 10 | 10 | 0 | 2R1 | 16 | 0 | 16 | 2C | | 12 | 12 | 0 |  |
|  | 2R2 | 14 | 8 | 6 |  |  |  |  | 2R2 | 20 | 0 | 20 |  |  |  |  | 2R2 | 23 | 0 | 23 |  | |  |  |  |  |

**Legend for Additional File 2.** . T – total number of mosquitoes exposed; S– number of mosquitoes surviving; M – number of dead mosquitoes; R1, R2 and R3 are different parallel replicate tests for each insecticide while 1R and 2R are two separate experiments; 1&2C are parallel replicate control tests for each insecticide while 1C and 2C are two separate experiments in parallel with 1R and 2R
