# Supplementary material for: Anopheline bionomics, insecticide resistance and transnational dispersion in the context of controlling a possible recurrence of malaria transmission in Jaffna city in northern Sri Lanka
Source: Parasit Vectors. 2020 Mar 30;13:156. doi: 10.1186/s13071-020-04037-x (PMC7106892; doi:10.1186/s13071-020-04037-x)
Supplement: Supplementary file 3 — Additional file 3: Alignment S1. DNA sequence alignments of NaV region. [file 13071_2020_4037_MOESM3_ESM.rtf]

Additional file 3: Alignment S1. DNA sequence alignments of NaV region 

             ....|....| ....|....| ....|....| ....|....| ....|....| 
                      10         20         30         40         50             
KF023569     TTGTGCGGGG AATGGATTGA ATCAATGTGG GACTGTATGC TTGTAGGTGA 
MK249733     TTGTGCGGGG AATGGATTGA ATCAATGTGG GACTGTATGC TTGTCGGTGA 
MK249732     TTGTGCGGGG AATGGATTGA ATCAATGTGG GACTGTATGC TTGTAGGTGA 
KF023553     TTGTGCGGGG AATGGATTGA ATCAATGTGG GACTGTATGC TTGTAGGTGA 
MK248685     CTGTGCGGCG AATGGATCGA GTCCATGTGG GACTGTATGC TTGTCGGTGA 
KJ636080     CTGTGCGGCG AATGGATCGA GTCCATGTGG GACTGTATGC TTGTCGGTGA 
Amino acid    L  C  G   E  W  I  E   S  M  W   D  C  M   L  V  G  V	

             ....|....| ....|....| ....|....| ....|....| ....|....| 
                      60         70         80         90        100            
KF023569     TGTGTCGTGC ATACCATTCT TCTTAGCTAC GGTAGTAATA GGAAATTTAG 
MK249733     TGTATCGTGC ATACCATTCT TCTTAGCTAC GGTAGTGATA GGAAATTTAG 
MK249732     TGTGTCGTGC ATACCATTCT TCTTAGCTAC GGTAGTAATA GGAAATTTCG 
KF023553     TGTGTCGTGC ATACCATTCT TCTTAGCTAC GGTAGTAATA GGAAATTTCG 
MK248685     CGTGTCGTGC ATACCCTTTT TCCTAGCTAC AGTAGTGATA GGAAATTTAG 
KJ636080     CGTGTCGTGC ATACCCTTTT TCCTAGCTAC AGTAGTGATA GGAAATTTAG 
Amino acid     D  S  C   I  P  F   F  L  A  T   V  V  I   G  N L/F  

             ....|
                   
KF023569     TGGTA
MK249733     TGGTA
MK249732     TGGTA
KF023553     TGGTA
MK248685     TCGTA
KJ636080     TCGTA
Amino acid   L  V


DNA sequence alignment of the fragment of NaV gene encompassing nucleotides corresponding to the codon 1014 in the present study:  Anopheles subpictus (MK249732), An. sundaicus  (MK249733), An. stephensi (MK248685) and other related GenBank entries: An. subpictus from India (KF023569 and KF023553) and An. stephensi from Iran (KJ636080). 
